# Semirecumbent Positioning During Anesthesia Recovery and Postoperative Hypoxemia: A Randomized Clinical Trial

**DOI:** 10.1001/jamanetworkopen.2024.16797

**Published:** 2024-06-28

**Authors:** Xinghe Wang, Kedi Guo, Jia Sun, Yuping Yang, Yan Wu, Xihui Tang, Yuqing Xu, Qingsong Chen, Si Zeng, Liwei Wang, Su Liu

**Affiliations:** 1 Jiangsu Province Key Laboratory of Anesthesiology, Xuzhou Medical University, Xuzhou, China; 2 Jiangsu Province Key Laboratory of Anesthesia and Analgesia Application Technology, Xuzhou Medical University, Xuzhou, China; 3 NMPA Key Laboratory for Research and Evaluation of Narcotic and Psychotropic Drugs, Xuzhou Medical University, Xuzhou, China; 4 Department of Anesthesiology, Affiliated Hospital of Xuzhou Medical University, Xuzhou, China; 5 Department of Anesthesiology, Xuzhou Central Hospital, Xuzhou, China; 6 Department of Anesthesiology, Sichuan Provincial People’s Hospital, University of Electronic Science and Technology of China, Chengdu, China

## Abstract

**Importance:**

The efficacy of a semirecumbent position (SRP) in reducing postoperative hypoxemia during anesthesia emergence is unclear despite its widespread use.

**Objective:**

To determine the differences in postoperative hypoxemia between patients in an SRP and a supine position.

**Design, Setting, and Participants:**

This randomized clinical trial was performed at a tertiary hospital in China between March 20, 2021, and May 10, 2022. Patients scheduled to undergo laparoscopic upper abdominal surgery under general anesthesia were enrolled. Study recruitment and follow-up are complete.

**Interventions:**

Patients were randomized to 1 of the following positions at the end of the operation until leaving the postanesthesia care unit: supine (group S), 15° SRP (group F), or 30° SRP (group T).

**Main Outcomes and Measures:**

The primary outcome was the incidence of postoperative hypoxemia in the postanesthesia care unit. Severe hypoxemia was also evaluated.

**Results:**

Out of 700 patients (364 men [52.0%]; mean [SD] age, 47.8 [11.3] years), 233 were randomized to group S (126 men [54.1%]; mean [SD] age, 48.2 [10.9] years), 233 to group F (122 men [52.4%]; mean [SD] age, 48.1 [10.9] years), and 234 to group T (118 women [50.4%]; mean [SD] age, 47.2 [12.1] years). Postoperative hypoxemia differed significantly among the 3 groups (group S, 109 of 233 [46.8%]; group F, 105 of 233 [45.1%]; group T, 76 of 234 [32.5%]; *P* = .002). This difference was statistically significant for groups T vs S (risk ratio [RR], 0.69 [95% CI, 0.55-0.87]; *P* = .002) and groups T vs F (RR, 0.72 [95% CI, 0.57-0.91]; *P* = .007), but not for groups F vs S (RR, 0.96 [95% CI, 0.79-1.17]; *P* = .78). Severe hypoxemia also differed among the 3 groups (group S, 61 of 233 [26.2%]; group F, 53 of 233 [22.7%]; group T, 36 of 234 [15.4%]; *P* = .01). This difference was statistically significant for groups T vs S (RR, 0.59 [95% CI, 0.41-0.85]; *P* = .005).

**Conclusions and Relevance:**

In this randomized clinical trial of SRP during anesthesia recovery in patients undergoing laparoscopic upper abdominal surgery, postoperative hypoxemia was significantly reduced in group T compared with group F or group S.

**Trial Registration:**

Chinese Clinical Trial Registry Identifier: ChiCTR2100045087

## Key Points

**Question:**

Does semirecumbent positioning during recovery from anesthesia reduce the incidence of postoperative hypoxemia?

**Findings:**

In this randomized clinical trial that included 700 adults undergoing laparoscopic upper abdominal surgery, a 30° semirecumbent position during recovery from anesthesia reduced the incidence of postoperative hypoxemia from 47% in a supine position to 32%.

**Meaning:**

These findings suggest that a 30° semirecumbent position during recovery from anesthesia may help prevent the development of postoperative hypoxemia.

## Teaser

This randomized clinical trial tests the efficacy and the optimal tilt angle of the semirecumbent position during anesthesia emergence in reducing postoperative hypoxemia among patients undergoing laparoscopic upper abdominal surgery.

## Introduction

Postoperative hypoxemia is common in patients recovering from noncardiac surgery, occurring in more than one-third of all cases.^1^ Severe hypoxemia can result in arrhythmias, abnormal blood pressure changes, and nervous system damage, all of which adversely affect patients’ postoperative recovery.^2,3,4^ Although oxygen administration can prevent and treat hypoxemia, many patients still experience hypoxia in the postanesthesia care unit (PACU).^5,6^

Numerous studies^7,8,9,10^ have investigated various ventilatory techniques aimed at enhancing postoperative pulmonary function, but the benefits of protective ventilation techniques may be lost during anesthesia emergence.^11^ Several other studies^12,13,14^ have also shown that intraoperative ventilation measures do not improve postoperative lung function. The lack of evidence demonstrating effective treatment of postoperative hypoxemia with oxygen therapy or protective ventilation techniques highlights the need to identify alternative strategies.

Patient positioning during anesthesia emergence is related to perioperative and postoperative complications.^15,16^ While there is no established consensus on the optimal patient position for extubation, the supine position is often favored by anesthesiologists due to its simplicity and ease of observation.^17^ However, the reduction in functional residual capacity associated with the supine position tends to promote airway closure and reduced gas exchange.^18,19^ In contrast, the semirecumbent position (SRP) has been shown to increase lung capacity by 10% to 15%, increase the functional residual capacity, and improve the range of motion of the diaphragm, resulting in improved lung expansion and gas exchange.^20,21^ However, to our knowledge, no studies have investigated whether SRP during anesthetic emergence may reduce the incidence of postoperative hypoxemia.

Accordingly, we performed a randomized clinical trial to test the efficacy and the optimal tilt angle of SRP during anesthesia emergence in reducing postoperative hypoxemia in a large sample of participants. We hypothesized that 30° SRP during anesthesia emergence would reduce the incidence of postoperative hypoxemia in adults undergoing laparoscopic upper abdominal surgery (LUAS).

## Methods

### Study Design

This single-center randomized clinical trial was approved by the ethics committee of the affiliated hospital of Xuzhou Medical University, Xuzhou, China. The trial protocol is available in Supplement 1. All participants involved provided written informed consent prior to their enrollment. The study followed the Consolidated Standards of Reporting Trials (CONSORT) reporting guideline.

### Patients

Patients 18 years or older with American Society of Anesthesiologists health status 1 to 3 and undergoing elective LUAS under general anesthesia were eligible to participate. Exclusion criteria were a body mass index (BMI; calculated as the weight in kilograms divided by the height in meters squared) of greater than 35, requirement of emergency surgery, any underlying lung disease, previous lung surgery, pneumothorax, pulmonary tuberculosis, pleural effusion, cases where intubation was expected to be difficult, respiratory failure, severe heart block, severe sinoatrial node dysfunction, congestive heart failure, pregnancy, and patient refusal. The dropout criteria included withdrawal of consent, cancellation of the procedure or the need to change to open surgery, admission to the intensive care unit (ICU), intraoperative hemodynamic instability, and/or other serious perioperative adverse events. Race and ethnicity were not collected.

### Intervention

At the end of the operation, patients were moved onto a transfer bed and then placed in a supine position (group S), a 15° SRP (group F), or a 30° SRP (group T) until leaving the PACU. After extubation, conventional oxygen therapy was administered.^22^ In patients who developed an oxygen saturation level by pulse oximeter (Spo_2_) of less than 90% for more than 1 minute, rescue mask ventilation was applied.^23^ A decision to reintubate was made by the anesthesiologist in the PACU if the ratio of Spo_2_/fraction of inspired oxygen (Fio_2_) to respiratory rate was less than 4.88.^24^ If there was evidence of hemodynamic instability, the intervention was cancelled by the anesthesiologist.

### Randomization and Masking

Randomization was performed using a computer-generated random numbers table with a block size of 6 and a 1:1:1 ratio. Numbers were allocated sequentially and sealed in opaque envelopes by 1 investigator (S.L.). The anesthesiologists opened the envelopes 10 minutes prior to the emergence procedure. Due to the nature of the intervention, full blinding of all study personnel was not feasible. Therefore, we only blinded postoperative data collectors and data analysts (including X.W., K.G., Y.Y., Y.W., Q.C., and S.Z.).

### Anesthesia Protocol

Continuous monitoring was conducted for all patients through the use of electrocardiography, noninvasive blood pressure measurements, bispectral index electrodes, Spo_2_ measurements, and end-tidal carbon dioxide measurements. Sufentanil citrate, propofol, and rocuronium bromide were used for intravenous anesthesia induction. Anesthesia was maintained with remifentanil hydrochloride and propofol. After intubation, an arterial catheter was placed in the radial artery for blood gas sampling and invasive blood pressure monitoring. Postoperative patient-controlled intravenous analgesia was administered (sufentanil citrate, 2 μg/kg, and tropisetron hydrochloride, 10 mg, diluted in 0.9% saline for a total solution volume of 100 mL) and was set at 0.5 mL/press with a 15-minute locking time. Moderate pain was treated with intravenous fentanyl citrate, 50 μg, and severe pain with intravenous fentanyl citrate, 100 μg. Once the Aldrete score was at least 9 of a possible 10, the patient was discharged back to the ward.^25^ The detailed anesthesia protocol is available in Supplement 1.

### Outcomes

The primary outcome was the incidence of postoperative hypoxemia in the PACU (defined as Spo_2_ <90% for more than 10 seconds or Pao_2_:Fio_2_ ratio <300 mm Hg). We also recorded the incidence of severe hypoxemia (Spo_2_ <85% for more than 10 seconds) and the time to first episode of hypoxemia. The secondary outcomes included airway rescue (defined as the need for mask noninvasive positive pressure ventilation or reintubation), coughing, extubation time, respiratory comfort, duration in the PACU, wound pain, and position-related adverse events (hypotension, arrhythmia, and some other adverse events). The patient’s vital signs (heart rate and mean arterial pressure) were recorded at 6 points: (1) the beginning of surgery, (2) the end of surgery, (3) immediately after adjusting position, (4) immediately prior to extubation, (5) 1 minute after extubation, and (6) when leaving the PACU. Dynamic lung compliance (Cdyn) and driving pressure were also recorded at 3 points: (1) when stitching, (2) 5 minutes after adjusting position, and (3) before extubation. Arterial blood gas analysis was performed at 3 points: (1) when stitching, (2) 5 minutes after adjusting position, and (3) 5 minutes post extubation.

### Sample Size Calculation

The sample size for the primary outcome of this study was calculated with continuity correction and was based on a ratio of exposed (combined 15° and 30° SRP) to unexposed (supine) groups of 2:1.^26,27^ According to our pilot study, the frequency of hypoxemia was 46% in the unexposed group, with a 26% relative (12% absolute) reduction in the exposed group. Assuming α = .05, a sample size of 620 was needed to give greater than 80% power to detect a decrease in the incidence of hypoxemia from 46% in the supine positioning group to 34% in the combined 15° and 30° SRP groups. Finally, we planned to recruit 700 patients to compensate for dropouts. The sample size calculation was conducted using PASS software, version 11.0 (NCSS).

### Statistical Analysis

Continuous variables were presented as mean (SD) or median (IQR), depending on whether the data were distributed normally or not. Categorical variables were presented as numbers (percentages). The normal distribution of data was evaluated using the Shapiro-Wilk test, and the Levene method was used to test the homogeneity of variance. Differences in repeated-measures variables were analyzed by repeated-measures analysis of variance. Continuous outcomes were analyzed with 1-way analysis of variance or Kruskal-Wallis test. Categorical variables were tested using a χ^2^ test or a Fisher exact test. The primary analyses were performed according to the intention-to-treat (ITT) principle. Modified ITT (mITT) and per-protocol (PP) analyses were also performed as sensitivity analyses for the primary outcome. Kaplan-Meier curves were used for the time to first experience hypoxemia. When an overall intervention effect was detected (*P* < .05), the Bonferroni correction for multiple pairwise comparisons was performed.

To handle missing data, multiple imputations by chained equations were used, assuming missing data are missing at random. As a post hoc sensitivity analysis, we used a logistic regression model simultaneously entering age, BMI, current smoking status, chronic pain needing opioids, Assess Respiratory Risk in Surgical Patients in Catalonia score,^28^ Charlson Comorbidity Index,^29^ surgery time, intraoperative opioid consumption, and the use of a nasogastric tube. R, version 4.1.1 (R Project for Statistical Computing), was used for statistical analysis, and analysis items with 2-sided *P* < .05 were considered statistically significant.

## Results

### Patient Characteristics and Perioperative Variables

**Figure 1.**
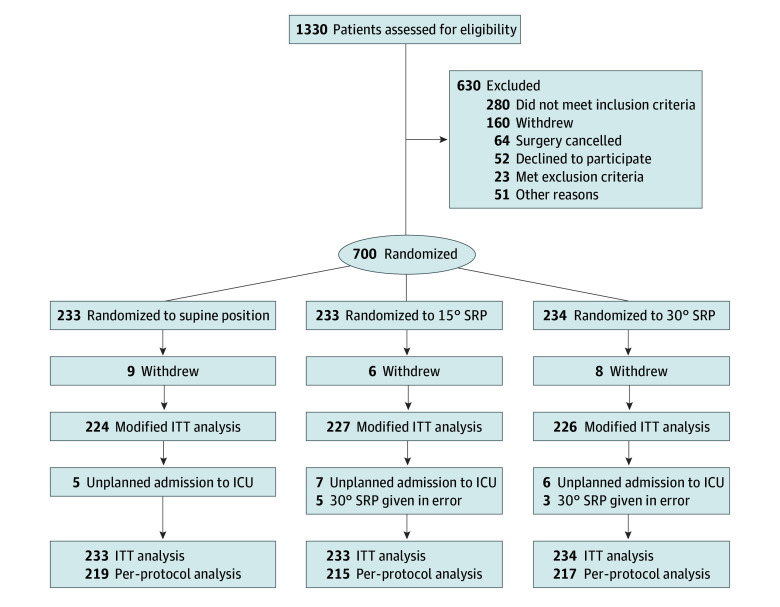
Study Flow Diagram Inclusion and exclusion criteria are described in the Patients subsection of the Methods section of the text. ICU indicates intensive care unit; ITT, intention-to-treat; and SRP, semirecumbent position.

Of a total of 700 patients (364 men [52.0%] and 336 women [48.0%]; mean [SD] age, 47.8 [11.3] years), 233 were randomized to group S (126 men [54.1%] and 107 women [45.9%]; mean [SD] age, 48.2 [10.9] years), 233 to group F (122 men [52.4%] and 111 women [47.6%]; mean [SD] age, 48.1 [10.9] years), and 234 to group T (116 men [49.6%] and 118 women [50.4%]; mean [SD] age, 47.2 [12.1] years) and included in the ITT analysis. Participant recruitment started on March 20, 2021, and the last participant completed the follow-up evaluation on May 10, 2022. Of the 1330 participants screened for eligibility, 700 were enrolled and randomized in the trial. Among these participants, 23 (3.3%) requested to withdraw; therefore, 677 were available for the mITT analysis. Of these, 26 patients (3.7%) had an unplanned ICU stay or received the incorrect intervention, and 651 were available for the PP analysis (Figure 1). The baseline characteristics and perioperative variables of participants are summarized in Table 1.

**Table 1.** Baseline Characteristics and Perioperative Variables^a^

Characteristic	Group S (n = 233)	Group F (n = 233)	Group T (n = 234)
Age, mean (SD), y	48.2 (10.9)	48.1 (10.9)	47.2 (12.1)
Sex			
Female	107 (45.9)	111 (47.6)	118 (50.4)
Male	126 (54.1)	122 (52.4)	116 (49.6)
BMI, mean (SD)	23.5 (2.7)	23.3 (2.9)	23.3 (2.8)
ASA health status			
1	73 (31.3)	70 (30.0)	73 (31.2)
2	130 (55.8)	134 (57.5)	129 (55.1)
3	30 (12.9)	29 (12.4)	32 (13.7)
ARISCAT score			
Low risk	62 (26.6)	64 (27.5)	67 (28.6)
Moderate risk	166 (71.2)	164 (70.4)	161 (68.8)
High risk	5 (2.1)	5 (2.1)	6 (2.6)
CCI			
0	146 (62.7)	141 (60.5)	143 (61.1)
1-2	60 (25.8)	67 (28.8)	63 (26.9)
3-4	22 (9.4)	19 (8.2)	21 (9.0)
≥5	5 (2.1)	6 (2.6)	7 (3.0)
Current smoker	33 (14.2)	34 (14.6)	32 (13.7)
Alcohol misuse	10 (4.3)	8 (3.4)	11 (4.7)
Hypertension	23 (9.9)	21 (9.0)	25 (10.7)
Long-term opioid use for chronic pain	13 (5.6)	12 (5.2)	13 (5.6)
Type 2 diabetes	13 (5.6)	11 (4.7)	15 (6.4)
Anesthesia time, mean (SD), h	4.5 (1.1)	4.5 (1.0)	4.4 (1.0)
Duration of surgery, mean (SD), h	3.7 (1.1)	3.7 (1.0)	3.6 (1.0)
Intraoperative opioid use, mean (SD), morphine equivalents	27.9 (7.2)	27.2 (7.9)	27.3 (8.2)
Tidal volume, mean (SD), mL/kg of predicted body weight^b^			
Start of surgery	7.0 (0.7)	7.1 (0.7)	7.1 (0.7)
End of surgery	7.0 (0.6)	7.1 (0.7)	7.1 (0.7)
PEEP, mean (SD), cm H_2_O			
Start of surgery	6.3 (0.9)	6.4 (0.8)	6.4 (0.9)
End of surgery	6.4 (0.9)	6.4 (0.8)	6.3 (0.9)
Plateau pressure, mean (SD), cm H_2_O			
Start of surgery	14.9 (3.2)	15.0 (3.5)	15.2 (3.6)
End of surgery	15.0 (3.0)	15.2 (3.2)	15.4 (3.0)
Fluid administered, median (IQR), mL			
Crystalloids	1950 (1750-2150)	2000 (1750-2200)	1900 (1750-2200)
Colloids	700 (600-840)	700 (600-800)	700 (600-850)
Blood loss, median (IQR), mL	350 (250-460)	360 (260-480)	355 (250-480)
Blood transfusion	24 (10.3)	22 (9.4)	24 (10.3)
Use of nasogastric tube	53 (22.7)	55 (23.6)	51 (21.8)

Abbreviations: ARISCAT, Assess Respiratory Risk in Surgical Patients in Catalonia; ASA, American Society of Anesthesiologists; BMI, body mass index (calculated as weight in kilograms divided by height in meters squared); CCI, Charlson Comorbidity Index; F, 15° semirecumbent position (SRP); PEEP, positive end-expiratory pressure; S, supine; T, 30° SRP.

^a^ Data are presented as No. (%) of patients unless otherwise indicated.

^b^ For men, 50 + 0.91 × (height in centimeters − 152.4); for women, 45.5 + 0.91 × (height in centimeters − 152.4).

### Primary Outcome

**Figure 2.**
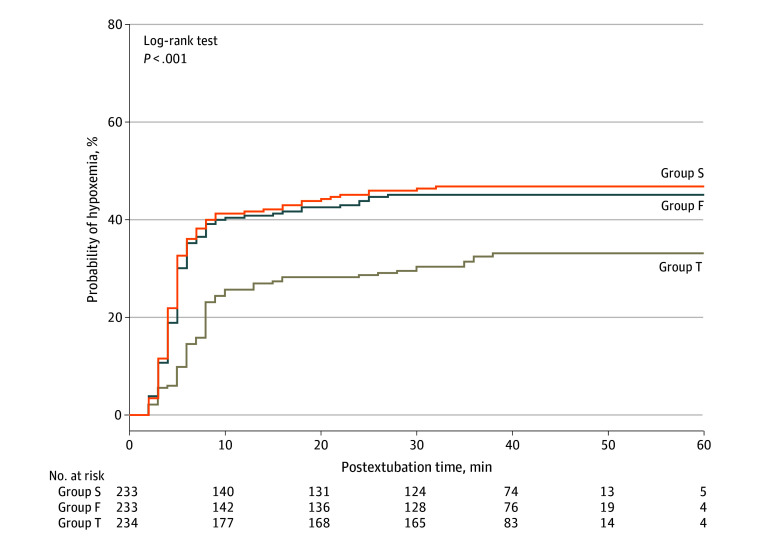
Kaplan-Meier Survival Analysis of Time to First Develop Hypoxemia F indicates 15° semirecumbent position (SRP); S, supine; and T, 30° SRP.

In the ITT analysis, postoperative hypoxemia occurred in 290 of 700 participants (41.4%). The incidence of hypoxemia was significantly different among the 3 groups (group S: 109 of 233 [46.8%]; group F: 105 of 233 [45.1%]; and group T: 76 of 234 [32.5%]; *P* = .002) (Table 2 and Figure 2). This difference was statistically significant for groups T vs S (risk ratio [RR], 0.69 [95% CI, 0.55-0.87]; *P* = .002) and groups T vs F (RR, 0.72 [95% CI, 0.57-0.91]; *P* = .007), but not for groups F vs S (RR, 0.96 [95% CI, 0.79-1.17]; *P* = .78) (Table 2). The mITT and PP analyses demonstrated similar results as the ITT analysis (Table 2). In addition, the incidence of severe hypoxemia was significantly different among the 3 groups (group S: 61 of 233 [26.2%]; group F: 53 of 233 [22.7%]; group T: 36 of 234 [15.4%]; *P* = .01) (Table 2). Pairwise comparisons showed this difference was statistically significant for groups T vs S (RR, 0.59 [95% CI, 0.41-0.85]; *P* = .005).

**Table 2.** Primary and Secondary Outcomes

Variable	Group^a^			*P* value	Group comparisons^b^					
					Group F vs S		Group T vs S		Group T vs F	
	S	F	T		RR or MD (95% CI)	*P* value	RR or MD (95% CI)	*P* value	RR or MD (95% CI)	*P* value
Hypoxemia										
ITT analysis	109/233 (46.8)	105/233 (45.1)	76/234 (32.5)	.002	0.96 (0.79 to 1.17)	.78	0.69 (0.55 to 0.87)	.002	0.72 (0.57 to 0.91)	.007
mITT analysis	107/224 (47.8)	103/227 (45.4)	74/226 (32.7)	.002	0.95 (0.78 to 1.16)	.67	0.69 (0.54 to 0.86)	.001	0.72 (0.57 to 0.91)	.008
PP analysis	102/219 (46.6)	98/215 (45.6)	72/217 (33.2)	.006	0.98 (0.80 to 1.20)	.91	0.71 (0.56 to 0.90)	.005	0.73 (0.57 to 0.92)	.01
Severe hypoxemia	61/233 (26.2)	53/233 (22.7)	36/234 (15.4)	.01	0.87 (0.63 to 1.20)	.85	0.59 (0.41 to 0.85)	.005	0.68 (0.46 to 0.99)	.05
Airway rescue	16/233 (6.9)	14/233 (6.0)	4/234 (1.7)	.02	0.88 (0.44 to 1.75)	.66	0.25 (0.08 to 0.73)	.01	0.28 (0.10 to 0.85)	.03
Coughing	47/233 (20.2)	39/233 (16.7)	25/234 (10.7)	.02	0.83 (0.57 to 1.22)	.40	0.53 (0.34 to 0.83)	.006	0.64 (0.40 to 1.02)	.08
Adverse events	0/233	0/233	0/234	NA	NA	NA	NA	NA	NA	NA
Extubation time, mean (SD), min	30.6 (14.1)	32.0 (14.4)	31.5 (14.4)	.57	NA	NA	NA	NA	NA	NA
Respiratory comfort score, mean (SD)^c^	6.7 (1.7)	7.4 (1.2)	7.8 (1.5)	<.001	0.7 (0.4 to 1.0)	<.001	1.1 (0.7 to 1.4)	<.001	0.4 (0.1 to 0.7)	.01
Duration in PACU, mean (SD), min	79.6 (16.5)	80.8 (18.4)	67.9 (17.6)	<.001	1.2 (−2.7 to 5.1)	.99	−11.7 (−15.6 to −7.8)	<.001	−13.0 (−16.9 to −9.1)	<.001

Abbreviations: F, 15° semirecumbent position (SRP); ITT, intention-to-treat; MD, mean difference; mITT, modified ITT; NA, not applicable; PACU, postanesthesia care unit; PP, per-protocol; RR, risk ratio; S, supine; T, 30° SRP.

^a^ Unless indicated otherwise, data are expressed as No./total No. (%) of patients.

^b^ For data expressed as No./total No. (%) of patients, values represent the RR (95% CI); for data expressed as mean (SD), values represent the MD (95% CI).

^c^ Scores range from 0 to 10, with higher scores indicating greater comfort.

### Secondary Outcomes

Airway rescue was required for 16 patients (6.9%) in group S, 14 (6.0%) in group F, and 4 (1.7%) in group T (*P* = .02). This difference was statistically significant for groups T vs S (RR, 0.25 [95% CI, 0.08-0.73]; *P* = .01) and groups T vs F (RR, 0.28 [95% CI, 0.10-0.85]; *P* = .03) (Table 2). The frequency of coughing also differed across groups (group S, 47 [20.2%]; group F, 39 [16.7%]; group T, 25 [10.7%]; *P* = .02) and was significantly less in groups T vs S (RR, 0.53 [95% CI, 0.34-0.83]; *P* = .006). The respiratory comfort score was significantly higher in group T compared with group S (mean difference, 1.1 [95% CI, 0.7-1.4]; *P* < .001) and with group F (mean difference, 0.4 [95% CI, 0.1-0.7]; *P* = .01). The mean difference in duration in PACU in group T was 11.7 (95% CI, −15.6 to −7.8) minutes shorter than in group S and 13.0 (95% CI, −16.9 to −9.1) minutes shorter than in group F (both *P* < .001) (Table 2). However, extubation time was similar among the 3 groups (Table 2). No other adverse events related to the SRP occurred during the time in PACU (Table 2).

The mean (SD) vital signs of all 3 groups fluctuated significantly 1 minute after extubation, but the heart rate (71.9 [5.6] beats/min [bpm]) and mean arterial pressure (98.7 [14.4] mm Hg) in group T were more stable than in groups S (78.5 [6.1] bpm and 104.7 [14.7] mm Hg; *P* < .001 for both) and F (75.4 [6.2] bpm and 102.1 [13.4] mm Hg; *P* < .001 and *P* = .01, respectively) (eFigure 1 in Supplement 2). The postoperative pain score at rest was similar among the groups at any given time point. However, at 3 points (5 minutes post extubation, 30 minutes post extubation, and before leaving the PACU), the mean (SD) pain score while coughing was significantly lower in group T (4.2 [1.3], 3.8 [1.3], and 3.1 [1.1], respectively) compared with group S (4.8 [1.5], 4.3 [1.6], and 3.7 [1.6], respectively; *P* < .001, *P* = .002, and *P* < .001, respectively) and group F (4.7 [1.4], 4.3 [1.5], and 3.8 [1.5], respectively; *P* = .001, *P* = .001, and *P* < .001, respectively) (eFigure 2 in Supplement 2).

### Pulmonary Function and Gas Exchange Parameters

As indicated in the eTable in Supplement 2, the pulmonary function and gas exchange parameters (Cdyn, driving pressure, Paco_2_, and Pao_2_:Fio_2_) were similar among the 3 groups during skin closure. However, Cdyn was statistically higher in group T than in group S (mean difference at 5 minutes after adjusting position, 3.2 [95% CI, 1.3-5.2] mL/cm H_2_O; mean difference before extubation, 3.7 [95% CI, 1.8-5.5] mL/cm H_2_O; *P* < .001 for both) or in group F (mean difference prior to extubation, 2.4 [95% CI, 0.6-4.2] mL/cm H_2_O; *P* = .005). At any point, Cdyn was similar between group F and group S. Driving pressure was statistically lower at 5 minutes after adjusting position in group T than in group S (mean difference, −2.7 [95% CI, −3.4 to −2.0] cm H_2_O) and group F (mean difference, −2.0 [95% CI, −2.7 to −1.2] cm H_2_O) and prior to extubation (mean difference, −2.2 [95% CI, −2.8 to −1.7] and −1.4 [95% CI, −2.0 to −0.9] cm H_2_O, respectively) (all *P* < .001). Driving pressure was statistically lower in group F compared with group S before extubation (mean difference, −0.8 [95% CI, −1.4 to −0.2] cm H_2_O; *P* = .003). At 5 minutes post extubation, Paco_2_ was statistically lower in group T than in group S (mean difference, −4.6 [95% CI, −6.1 to −3.2] mm Hg) and group F (mean difference, −3.2 [95% CI, −4.7 to −1.7] mm Hg) (both *P* < .001). An improved Pao_2_:Fio_2_ ratio was observed in group T compared with group S at 5 minutes after adjusting position (mean difference, 25.4 [95% CI, 17.0- 33.8] mm Hg) and 5 minutes post extubation (mean difference, 27.7 [95% CI, 20.7-34.7] mm Hg) and compared with group F at 5 minutes after adjusting position (mean difference, 17.9 [95% CI, 9.4- 26.3] mm Hg) and 5 minutes post extubation (mean difference, 22.8 [95% CI, 15.8-29.8] mm Hg) (all *P* < .001).

### Sensitivity Analysis

**Figure 3.**
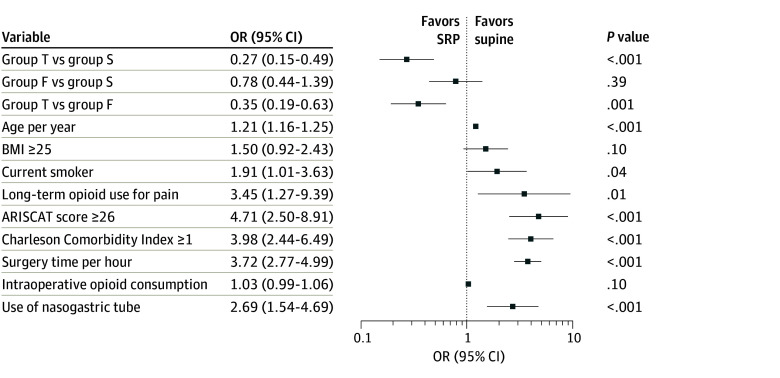
Post Hoc Sensitivity Analysis Using Multiple Logistic Regression ARISCAT indicates Assess Respiratory Risk in Surgical Patients in Catalonia (≥26 indicates intermediate-to-high risk for postoperative pulmonary complications); BMI, body mass index (calculated as weight in kilograms divided by height in meters squared); F, 15° semirecumbent position (SRP); S, supine; and T, 30° SRP. The x-axis was log^10^ transformed.

The beneficial effect of a 30° SRP on postoperative hypoxemia remained after analysis. We used variables that may influence postoperative hypoxemia, including age, BMI, current smoking status, chronic pain requiring opioids, Assess Respiratory Risk in Surgical Patients in Catalonia score, Charlson Comorbidity Index, surgery time, intraoperative opioid consumption, and use of nasogastric tube (Figure 3).

## Discussion

In this randomized clinical trial, a 30° SRP at anesthesia emergence was associated with a lower incidence of hypoxemia after extubation among patients undergoing LUAS. Anesthesia emergence involves a complex transition from a controlled to an uncontrolled situation. Anatomical and physiological changes are compounded by constraints such as time pressures that ultimately contribute to a situation that can be more challenging than tracheal intubation for the anesthesiologist.^30^ Major airway complications occurred during anesthesia emergence in approximately one-third of the reported cases relating to anesthesia, with hypoxemia being a common occurrence.^3,31^ Although oxygen administration and incentive spirometry are effective in treating most cases of postoperative hypoxemia, respiratory failure may occur early in the postoperative course, requiring reintubation and mechanical ventilation in 8% to 10% of patients, thus increasing morbidity and mortality and prolonging ICU and hospital stay.^32,33,34,35^ An essential component for adequate gas exchange and oxygenation of arterial blood is matching of the ventilation-perfusion ratio. A mismatch of ventilation and perfusion results in hypoxemia.^36^ Body position directly impacts ventilation and perfusion matching and can therefore influence arterial oxygen levels.^37^ However, evidence for the effect of body positioning on postoperative hypoxemia, particularly during emergence from anesthesia, is scarce. Several studies^38,39,40,41^ have demonstrated that prone and lateral positions could improve pulmonary function and oxygenation in patients undergoing mechanical ventilation. However, the populations studied were mostly those with severe lung disease or children, and the observation period was either intraoperative or in the ICU. Additionally, it may not be practical or convenient to adjust patients into a prone or lateral position in the PACU. In this study, patients were placed in an SRP immediately after surgery until leaving the PACU. The 30° SRP reduced the risk of postoperative hypoxemia. There was no evidence, however, to support that a 15° SRP would have a significant impact on postoperative hypoxemia. The 15° angle may be insufficient to bring about the proposed physiological changes that may occur at the 30° position. As a direct benefit of the reduced incidence of hypoxemia, the number of patients requiring airway rescue was similarly significantly reduced in group T.

A possible mechanism by which the 30° SRP reduced the incidence of postoperative hypoxemia is that, at the 30° position, the diaphragm moves downward, which in turn reduces the work of breathing and allows lung volumes and ventilation to increase, promoting lung dilation.^42,43^ All these changes would improve oxygenation and increase oxygen saturation. As reflected in our study, the 30° SRP is associated with improved lung function and gas exchange. A second possible mechanism is related to postoperative pain, as this is an important factor in inhibiting breath. A 30° SRP could reduce abdominal wall tension by shortening the linear distance between xiphoid and pubic, which in turn could help reduce postoperative pain. This is demonstrated by the fact that the pain scores while coughing were lower in the 30° group. Less postoperative pain would ultimately allow patients to breathe in a larger tidal volume, resulting in a reduced incidence of hypoxemia. Both the proposed physiological respiratory changes and reduced pain associated with the 30° SRP would also explain the increased respiratory comfort scores associated with the 30° position observed in this study.

Careful attention was paid to hemodynamic changes and cardiovascular safety, given the possibility of transient hypotension in the SRP. However, we found that the 30° SRP did not cause significant changes in hemodynamic parameters. Thomas et al^44^ also found that SRP did not result in significant changes to hemodynamic parameters. Interestingly, the vital signs of the 30° SRP group in this study were more stable than those of the other position groups immediately after extubation. This may be because in a supine position, the angle between the tracheal tube and the airway is close to vertical, whereas in the 30° SRP, this angle can be increased, resulting in lighter stimulation to the airway during extubation. Furthermore, Ling et al^45^ also found that bronchoscopy performed in the SRP resulted in less cough and improved patient comfort. In addition to its advantages for patients, our study identified that a 30° SRP during anesthesia emergence significantly reduced the PACU stay duration. This has the potential to enhance PACU operational efficiency and mitigate bed shortages. The efficacy demonstrated here of using the 30° SRP during anesthesia emergence, together with the low cost and effort required in its implementation and lack of significant adverse effects, makes it difficult to argue against using this simple technique to reduce postoperative hypoxemia and improve patient comfort.

### Limitations

There are some limitations to this study. First, we observed only 15° and 30° SRP; it is unclear whether a higher angle would provide further benefit. Second, full blinding of all study personnel was not feasible. Third, the main objective of this study was to observe the efficacy of SRP in reducing postoperative hypoxemia; therefore, we did not investigate other possible mechanisms associated with this positioning technique.

## Conclusions

In this randomized clinical trial, the 30° SRP during anesthesia emergence significantly reduced the incidence of postoperative hypoxemia in patients undergoing LUAS compared with the supine position. Furthermore, it reduced the occurrence of airway rescue and coughing, shortened PACU stay, and could enhance PACU operating efficiency.

## Notes

Supplement 1.
Trial Protocol

Supplement 2. **eTable.** Pulmonary Function and Gas Exchange Parameters of the Patients **eFigure 1.** Vital Signs of the Patients **eFigure 2.** Postoperative Pain of the Patients

Supplement 3.
Data Sharing Statement
