# Supplementary material for: Semirecumbent Positioning During Anesthesia Recovery and Postoperative Hypoxemia: A Randomized Clinical Trial
Source: JAMA Netw Open. 2024 Jun 28;7(6):e2416797. doi: 10.1001/jamanetworkopen.2024.16797 (PMC11214118; doi:10.1001/jamanetworkopen.2024.16797)
Supplement: Supplement 1. — Trial Protocol [file jamanetwopen-e2416797-s001.pdf]

Clinical Study Protocol

TITLE:

The effects of different degrees of semi-recumbent position during anesthesia emergence on postoperative hypoxemia in patients undergoing laparoscopic upper abdominal surgery

A Randomized Controlled, Clinical Trial

PRINCIPAL INVESTIGATOR: Xinghe Wang, MD, Kedi Guo, MD, Jia Sun, MD, Liwei Wang, MD, Su Liu, PhD.

PROTOCOL SYNOPSIS

Title: The effects of different degrees of semi-recumbent position during anesthesia emergence on postoperative hypoxemia in patients undergoing laparoscopic upper abdominal surgery

Study Type: A prospective, single-center, Randomized Controlled, Clinical Trial

Corresponding Author: Su Liu, Department of Anesthesiology, Affiliated Hospital of Xuzhou Medical University, Xuzhou, Jiangsu, China. Liwei Wang, Department of Anesthesiology, Xuzhou Central Hospital, Xuzhou, Jiangsu, China.

Study Center: Affiliated Hospital of Xuzhou Medical University

Ethics: Approved by the Ethics Committee of the Affiliated Hospital of Xuzhou Medical University (XYFY2021- KL090-01).

Trial Registration: www.chictr.org.cn (ChiCTR2100045087)

CONTENTS

I. STUDY OBJECTIVES 3
II. BACKGROUND 4
A. Postoperative hypoxemia 4
B. Prevention and treatment of postoperative hypoxemia 4
C. Preliminary Studies 5
III. METHODS 6
A. Recruiting Methods 6
B. Inclusion Criteria 7
C. Exclusion Criteria 7
D. Consent Procedure 7
E. Randomization and Blinding 7
F. Sample Size Calculation 8
G. Statistical Analysis 8
H. Intervention 8
I. Study Procedures 10
J. Measurements and Endpoints 11
IV. DATE AND SAFETY MONITORING 14

V. FUNDING 15
VI. INFORMATION CONFIDENTIALITY 16
VII. LITERATURE CITED 17

I. STUDY OBJECTIVES

Annually 313 million surgical procedures are performed worldwide.[1] However, few studies have explored the anesthesia emergence position in patients with general anesthesia. We aimed to investigate the efficacy and safety of different degrees of semi-recumbent position for anesthesia emergence.

II. BACKGROUND

A. Postoperative hypoxemia

Previous studies reported that hypoxemia, one of the most meaningful factors associated with poor patient outcomes, occurs in 10% to 50 % of patients after surgery, depending on the surgical population, definition of hypoxemia, and monitoring methods.[2,3] Severe hypoxemia can result in arrhythmias, abnormal blood pressure changes, and nervous system damage, all of which adversely affect patients' postoperative recovery.

B. Prevention and treatment of postoperative hypoxemia

Although oxygen therapy can prevent and treat hypoxemia, many patients will still develop hypoxia in the PACU even with prophylactic oxygen.[4,5] There have also been many studies regarding ventilatory techniques to improve postoperative lung function, but the benefits of protective ventilation techniques may be lost during emergence

process. Whalen et al.[6] found that recruitment maneuvre and the application of positive end-expiratory pressure (PEEP) improved intraoperative oxygenation, but the effect dissipated promptly during emergence process. Neither oxygen therapy nor protective ventilation techniques can completely eliminate postoperative hypoxemia, which means there is still room for improvement.

C. Preliminary Studies

Currently, only a few studies have focused on the position during anesthesia emergence. Although there is no evidence to support a universal patient position for extubation, most anesthesiologists prefer to place patients in supine position. This is because it is simple, and enables easy observation. However, the reduction in functional residual capacity (FRC) caused by the supine position tends to promote airway closure and reduce gas exchange.[7,8] Although, there is a growing preference for anesthesia emergence in semi-recumbent position. However, there is still a lack of reliable clinical evidence to demonstrate its efficacy, safety, and appropriate tilt angle. Therefore, we hypothesized that putting patients undergoing laparoscopic upper abdominal surgery in a semi-recumbent position during anesthesia emergence can reduce the incidence of postoperative hypoxemia.

III. METHODS

This is a prospective randomized, controlled trial that has been registered in the Chinese Clinical Trial Registry (ChiCTR2100045087) and has been approved by the Ethics Committee of the Affiliated Hospital of Xuzhou Medical University (XYFY2021- KL090-01). This report adheres to the CONSORT guidelines. The study was conducted according to the guidelines of the Declaration of Helsinki with Good Clinical Practice. All participants provided written informed consent before enrolment.

A. Recruiting Methods

Potential participants to the study were identified from the elective surgery list. Patients aged ≥18 years, ASA I to III, and undergoing elective laparoscopic upper abdominal surgery under general anesthesia were eligible to participate. The study will involve the use of protected health information. The study site will gain

permission from each subject to use their protected health information by written authorization. All subjects will be identified by the anesthesiologist.

B. Inclusion Criteria

Patients aged ≥18 years, ASA I to III, and undergoing elective laparoscopic upper abdominal surgery under general anesthesia were eligible to participate.

C. Exclusion Criteria

Body mass index (BMI)>35 $kg \cdot m^{-2}$, emergency surgery, underlying lung disease, previous lung surgery, pneumothorax, pulmonary tuberculosis, pleural effusion, expectation of difficult intubation, respiratory failure, severe heart block (II or III atrioventricular block, double-bundle branch block), severe sinoatrial node dysfunction, congestive heart failure, pregnancy, and patient's refusal.

D. Consent Procedure

All potential subjects that are identified by the chief anesthetist and/or designee that meet the inclusion/exclusion criteria will be given the opportunity to participate. Guardians/patients will be given the consent/assent during the screening visit. They will be given the opportunity to review the consent/assent and ask questions about the study. Guardians/patients will be asked to summarize in their own words what participation in this research study involves and that they are comfortable with the risks and benefits of participating in the research study. Any additional questions they have will also be answered by the investigator prior to signing the consent/assent. Once the consent/assent form is signed, a signed and dated copy of the authorization form will be provided to the subject and another copy placed in the participant's medical record.

E. Randomization and Blinding

Randomization was performed using a computer-generated random numbers table with a block size of 6 and a 1:1:1 ratio after stratification according to the type of surgery and age. Allocation was sequentially numbered and sealed in opaque

envelopes by the corresponding author. The anesthesiologists opened the envelopes 10 min before the emergence procedure. Because of the nature of the intervention, full blinding of all study personnel was not feasible. However, to minimize bias, we attempted to blind patients, practitioners (where possible), research assistants, and data analysts. It was not feasible to blind the PACU anesthesiologists and nurses. To achieve patient blinding, during the informed consent process, we described all interventions as anesthesia emergence positions, and we explained that depending on group allocation, patients may receive different emergence positions during anesthesia emergence. We additionally performed an analysis of blinding success of patients using Bang's Blinding Index.[9]

F. Sample Size Calculation

The sample size for the primary outcome of this study was calculated with continuity correction, and was based on a ratio of exposed (combined 15-degree and 30-degree SRP) to unexposed (supine positioning) groups of 2:1.[10,11] According to our pilot study, the frequency of hypoxaemia was 46% in the unexposed group, with a 26% relative (12% absolute) reduction in the exposed group. Assuming a two-tailed type I error rate of 5%, a sample size of 620 was needed to give greater than 80% power to detect a decrease in the incidence of hypoxaemia from 46% in the supine positioning group to 34% in the combined 15-degree and 30-degree SRP groups. Finally, we planned to recruit 700 patients to compensate for dropouts. The sample size calculation was conducted using the PASS 11.0 software (NCSS LLC).

G. Statistical Analysis

Continuous variables were presented as mean (SD) or median (interquartile range, [IQR]), depending on whether the data was distributed normally or not. Categorical variables were presented as a number (percentage). The normal distribution of data was evaluated using the Shapiro–Wilk test, and the Levene method was used to test the homogeneity of variance. Differences in repeated measures variables were analysed by repeated-measures analysis of variance. Continuous outcomes were

analysed with one-way analysis of variance (ANOVA) or Kruskal-Wallis test. Categorical variables were tested using χ2 test or Fisher’s exact test. The primary analyses were performed according to the intention-to-treat (ITT) principle. Modified ITT (mITT) and per-protocol (PP) analyses were also performed as sensitivity analyses for the primary outcome. Results of primary and secondary outcomes were reported as relative risk (RR) or between-group difference with 95 % CI, the absolute risk reduction (ARR) and Number needed to Treat (NNT) were also calculated, with 95% CI. Kaplan-Meier curves were used for the time to first experience hypoxaemia. When an overall intervention effect was detected ($P<0.05$), the Bonferroni correction for multiple pairwise comparisons would be performed. To handle missing data, multiple imputations by chained equations was used, assuming missing data are missing at random. Imputation for continuous data was performed by predictive mean matching, logistic regression for binary variables. The number of imputations was 5. Convergence of the multiple imputation algorithm and the results of the imputation were checked. Imputations were performed with the R MICE package (J Stat Software. 2011;45(3):1–67.). Analyses were then performed on each of the imputed data sets and pooled according to Rubin’s rules. As a post hoc sensitivity analysis, we used a logistic regression model simultaneously entering: age, BMI, current smoking status, chronic pain needing opioids, ARISCAT score (Assess Respiratory Risk in Surgical Patients in Catalonia),[12] CCI (Charlson Comorbidity Index),[13] surgery time, intraoperative opioid consumption, and the use of a nasogastric tube. R version 4.1.1 was used for statistical analysis, and analysis items with 2-sided $P<0.05$ were considered statistically significant.

H. Intervention

At the end of operation, patients were transferred from operating table to transfer bed, and their positions were adjusted according to the groups.

supine position group (group S): Patients were placed in supine position from the end of surgery to leaving PACU.

15-degree semi-recumbent position group (group F): Patients were placed in 15-

degree semi-recumbent position from the end of surgery to leaving PACU.

30-degree semi-recumbent position group (group T): Patients were placed in 30-degree semi-recumbent position from the end of surgery to leaving PACU.

The inclination angle was set precisely by an inclinometer (GeeLii 55157, Shanghai GeeLii International Trade Co., China)

I. Study Procedures

i. Preoperative assessment

During the preoperative assessment, we collected baseline data include demographic characteristics, medical history, pain management history and ARISCAT scores, as well as CCI.

ii. Perioperative management

Continuous monitoring was conducted for all patients through the use of electrocardiograms, noninvasive blood pressure measurements, bispectral index (BIS) electrodes (monitoring depth of anaesthesia), pulse oxygen saturation (SpO_2) measurements, and end-tidal CO_2 ($ETCO_2$) measurements. Sufentanil 0.5 μg/kg, propofol 1.5 to 2.0 mg/kg, and rocuronium 0.8 mg/kg were used for anaesthesia induction. Anaesthesia was maintained with remifentanil 0.1 to 0.3 μg/kg/min and propofol 4 to 12 mg/kg/h. After intubation, an arterial catheter was placed in the radial artery for blood gas sampling and invasive blood pressure monitoring. BIS value between 40 and 60 were maintained by adjusting the dose of propofol. The respiratory rate was initially set at 12 breaths/min and further adjusted to maintain end-tidal carbon dioxide pressure between 35 and 45 mmHg. Mechanical ventilation was maintained in a volume-controlled mode. Lung protective ventilation was used during surgery (the combination of tidal volume 8 ml/kg predicted body weight or lower and PEEP 5 cm H_2O or higher, with a recruitment manoeuvre, fraction of inspired oxygen 60%) in all patients. Mean arterial pressure was maintained within ±20% of the baseline. Hypotension was treated with phenylephrine 40 μg, ephedrine 5 mg, or a fluid bolus; episodes of hypertension were managed by increasing the propofol or remifentanil infusion rate. If the heart rate was less than 40/min, atropine 0.5 mg (i.v.)

was administered. The type and volume of fluid infusion were determined by the anesthesiologist. Rocuronium was continuously infused at a rate of 0.3 to 0.8 mg/kg/h and stopped approximately 40 minutes before the end of surgery. Postoperative patient-controlled intravenous analgesia (PCIA) was administered (sufentanil 2 μg/kg, tropisetron 10 mg, 0.9% saline diluted, the total volume of the solution is 100 ml.) and was set at 0.5 ml/press with a 15 minutes locking time. Pain scores (Visual Analogue Scale, VAS score) were assessed by nurses in the PACU. Moderate pain (VAS score 4-6) was treated with hydromorphone 0.25 mg, and severe pain (VAS score 7-10) with hydromorphone 0.5 mg, with an aim to reduce pain scores to less than 4. Once the Aldrete score was greater than or equal to 9 out of 10, the patient was discharged back to the ward.

Prior to extubation, all patients underwent suctioning, with no further stimulation being applied until spontaneous movement was observed. Neuromuscular blockade was reversed using intravenous neostigmine 40 to 60 μg/kg combined with atropine 0.5 mg. Prior to extubation the following criteria had to be met: complete reversal of neuromuscular blockade (a Train-Of-Four ratio greater than 0.90); patient fully awake; and breathing adequately.

After extubation, conventional oxygen therapy (low-flow oxygen (≤15 l/min) delivered either by Nasal cannula, FiO_2 0.35, oxygen flow 3.5 l/min; or Venturi mask, FiO_2 0.35, oxygen flow 9 l/min) was administered.[14] The recovery nurses did not intervene in the patient's breathing before the onset of hypoxemia, and only when hypoxemia occurred did the recovery nurses correct the hypoxia by encouraging the patient to take deep breaths and increasing the FiO_2. In patients who developed SpO_2 < 90% more than 1 minutes, rescue mask ventilation was applied.[15] A decision to re-intubate was made by the anaesthetist in the PACU if the ROX index (defined as the ratio of pulse oximetry/fraction of inspired oxygen to respiratory rate) was less than 4.88.[16] If there was evidence of heamodynamic instability, the intervention was cancelled by the anaesthetist.

J. Measurements and Endpoints

The primary outcome was the incidence of postoperative hypoxaemia in the PACU (defined as SpO_2 < 90% for more than 10 seconds or PaO_2:FiO_2 ratio below 300 mmHg). We also recorded the incidence of severe hypoxaemia (SpO_2 <85% for more than 10 seconds) and the time to first episode of hypoxaemia. Patients were continuously monitored real-time vital signs in the PACU using a Mindray BeneVision N15 monitor (Shenzhen Mindray Bio-Medical Electronics Co., Ltd.). In this study, data collection was performed by one-on-one researchers in the PACU, review of the monitor's database by the data collector when the patient leaving the PACU. To avoid pulse oximeter probe dislodging the finger or having it only partially on the finger, we used disposable finger probes that stick to the finger like a Band-Aid for continuous monitoring (Disposable SpO_2 Sensor, Shenzhen Mindray Bio-Medical Electronics Co., Ltd.).

The secondary outcomes included:

airway rescue (defined as the need for mask noninvasive positive pressure ventilation, or re-intubation);

respiratory symptoms (coughing);

extubation time (defined as the time from end of surgery to extubation);

respiratory comfort (before leaving PACU, graded using a numerical rating scale, ranging from 0 to 10, higher score means more comfort);

duration of stay in the PACU;

wound pain (VAS score measured at rest, while coughing at 5 and 30 minutes after extubation, before leaving PACU, and 24 hours post operation);

and position-related adverse events (hypotension, arrhythmia, and some other adverse events).

The patient's vital signs (heart rate and mean arterial pressure) were recorded at six time points: (1) the beginning of surgery (T0); (2) the end of surgery (T1); (3) immediately after adjusting position (T2); (4) immediately prior to extubation (T3); (5) 1 min after extubation (T4); (6) when leaving the PACU (T5).

Dynamic lung compliance (Cdyn) and driving pressure (△P) were also recorded at

three time points: (1) when stitching; (2) 5 minutes after adjusting position; and (3) before extubation.

Arterial blood gas analysis was performed at three time points: (1) when stitching; (2) 5 minutes after adjusting position; (3) 5 minutes post-extubation.

IV. DATE AND SAFETY MONITORING

Clinical research will formulate a corresponding data safety monitoring plan according to the size of the risk. In the implementation stage of clinical research, record all adverse events in detail, handle and track them properly until they are properly resolved or the condition is stable, and report serious adverse events and unexpected events to the ethics committee, competent authoritiesn and drug regulatory authorities in a timely manner as required; The principal investigator will periodically conduct a cumulative review of all adverse events, and if necessary, hold an investigator meeting to assess the risks and benefits of the study; We will arrange independent data monitors to monitor the research data, and high-risk studies will establish an independent data safety monitoring committee to monitor the accumulated safety data and efficacy data to make a decision on whether to continue the study.

V. FUNDING

This study was funded by Natural Science Research Fund of Higher Education Institutions in Jiangsu Province (22KJA320007); National Natural Science Foundation of China (82301414); Sichuan Provincial Science and Technology Program (2022YFS0301); Medical Research Project of Sichuan Province (S20025). The funders had no role in the design and conduct of the study; collection, management, analysis, and interpretation of the data; preparation, review, or approval of the manuscript; and decision to submit the manuscript for publication.

VI. INFORMATION CONFIDENTIALITY

Medical records will be kept in the hospital, the investigators and ethics committees will be allowed to access the patient's medical records. Any public reporting of the results of this study will not disclose the patient's personal identity.

VII. LITERATURE CITED

1. Weiser TG, Haynes AB, Molina G, et al. Estimate of the global volume of surgery in 2012: an assessment supporting improved health outcomes. *Lancet*. Apr 27 2015;385 Suppl 2:S11. doi:10.1016/s0140-6736(15)60806-6

2. Arozullah AM, Daley J, Henderson WG, Khuri SF. Multifactorial risk index for predicting postoperative respiratory failure in men after major noncardiac surgery. The National Veterans Administration Surgical Quality Improvement Program. *Ann Surg*. Aug 2000;232(2):242-53. doi:10.1097/00000658-200008000-00015

3. Lawrence VA, Dhanda R, Hilsenbeck SG, Page CP. Risk of pulmonary complications after elective abdominal surgery. *Chest*. Sep 1996;110(3):744-50. doi:10.1378/chest.110.3.744

4. Russell GB, Graybeal JM. Hypoxemic episodes of patients in a postanesthesia care unit. *Chest*. Sep 1993;104(3):899-903. doi:10.1378/chest.104.3.899

5. Scuderi PE, Mims GR, 3rd, Weeks DB, Harris LC, Lipscomb L, James RL. Oxygen administration during transport and recovery after outpatient surgery does not

prevent episodic arterial desaturation. *J Clin Anesth*. Jun 1996;8(4):294-300. doi:10.1016/0952-8180(96)00038-4

6. Whalen FX, Gajic O, Thompson GB, et al. The effects of the alveolar recruitment maneuver and positive end-expiratory pressure on arterial oxygenation during laparoscopic bariatric surgery. *Anesth Analg*. Jan 2006;102(1):298-305. doi:10.1213/01.ane.0000183655.57275.7a

7. Smith LJ, Macleod KA, Collier GJ, et al. Supine posture changes lung volumes and increases ventilation heterogeneity in cystic fibrosis. *PLoS One*. 2017;12(11):e0188275. doi:10.1371/journal.pone.0188275

8. Joosten SA, Landry SA, Sands SA, et al. Dynamic loop gain increases upon adopting the supine body position during sleep in patients with obstructive sleep apnoea. *Respirology*. Nov 2017;22(8):1662-1669. doi:10.1111/resp.13108

9. Kolahi J, Bang H, Park J. Towards a proposal for assessment of blinding success in clinical trials: up-to-date review. *Community Dent Oral Epidemiol*. Dec 2009;37(6):477-84. doi:10.1111/j.1600-0528.2009.00494.x

10. Avidan MS, Maybrier HR, Abdallah AB, et al.

Intraoperative ketamine for prevention of postoperative delirium or pain after major surgery in older adults: an international, multicentre, double-blind, randomised clinical trial. *Lancet*. Jul 15 2017;390(10091):267-275. doi:10.1016/S0140-6736(17)31467-8

11. Shi W, Chen Y, Zhang MQ, Che GW, Yu H. Effects of methylprednisolone on early postoperative pain and recovery in patients undergoing thoracoscopic lung surgery: A randomized controlled trial. *J Clin Anesth*. Dec 2021;75:110526. doi:10.1016/j.jclinane.2021.110526

12. Canet J, Gallart L, Gomar C, et al. Prediction of postoperative pulmonary complications in a population-based surgical cohort. *Anesthesiology*. Dec 2010;113(6):1338-50. doi:10.1097/ALN.0b013e3181fc6e0a

13. Charlson ME, Pompei P, Ales KL, MacKenzie CR. A new method of classifying prognostic comorbidity in longitudinal studies: development and validation. *J Chronic Dis*. 1987;40(5):373-83. doi:10.1016/0021-9681(87)90171-8

14. Zhu Y, Yin H, Zhang R, Ye X, Wei J. High-flow nasal cannula oxygen therapy versus conventional oxygen

therapy in patients after planned extubation: a systematic review and meta-analysis. *Crit Care*. May 17 2019;23(1):180. doi:10.1186/s13054-019-2465-y

15. Jeong H, Tanatporn P, Ahn HJ, et al. Pressure Support versus Spontaneous Ventilation during Anesthetic Emergence-Effect on Postoperative Atelectasis: A Randomized Controlled Trial. *Anesthesiology*. Dec 1 2021;135(6):1004-1014. doi:10.1097/ALN.0000000000003997

16. Roca O, Caralt B, Messika J, et al. An Index Combining Respiratory Rate and Oxygenation to Predict Outcome of Nasal High-Flow Therapy. *Am J Respir Crit Care Med*. Jun 1 2019;199(11):1368-1376. doi:10.1164/rccm.201803-0589OC
