# Supplementary material for: Semirecumbent Positioning During Anesthesia Recovery and Postoperative Hypoxemia: A Randomized Clinical Trial
Source: JAMA Netw Open. 2024 Jun 28;7(6):e2416797. doi: 10.1001/jamanetworkopen.2024.16797 (PMC11214118; doi:10.1001/jamanetworkopen.2024.16797)
Supplement: Supplement 2. — eTable. Pulmonary Function and Gas Exchange Parameters of the Patients eFigure 1. Vital Signs of the Patients eFigure 2. Postoperative Pain of the Patients [file jamanetwopen-e2416797-s002.pdf]

Supplementary Online Content

Wang X, Guo K, Sun J, et al. Semirecumbent positioning during anesthesia recovery and postoperative hypoxemia: a randomized clinical trial. *JAMA Netw Open*. 2024;7(6):e2416797. doi:10.1001/jamanetworkopen.2024.16797

eTable. Pulmonary Function and Gas Exchange Parameters of the Patients
eFigure 1. Vital Signs of the Patients
eFigure 2. Postoperative Pain of the Patients

This supplementary material has been provided by the authors to give readers additional information about their work.

© 2024 Wang X et al. *JAMA Network Open*.

eTable. Pulmonary Function and Gas Exchange Parameters of the Patients

Variable	Group S (n=233)	Group F (n=233)	Group T (n=234)	*P*	Group F vs S MD (95%CI)	*P*	Group T vs S MD (95%CI)	*P*	Group T vs F MD (95%CI)	*P*
**Cdyn (ml/cmH_2O)**										
When stitching	53.3 (7.8)	53.4 (7.4)	54.0 (8.0)	.54						
5min after adjusting position	54.5 (8.2)	55.7 (8.8)	57.8 (8.7)	<.001	1.1(-0.8-3.1)	.45	3.2(1.3-5.2)	<.001	2.1(0.2-4.0)	.03
Before extubation	56.5 (7.8)	57.8 (8.0)	60.2 (8.8)	<.001	1.3(-0.6-3.1)	.29	3.7(1.8-5.5)	<.001	2.4(0.6-4.2)	.005
**△P (cmH_2O)**										
When stitching	14.2 (2.9)	13.9 (2.9)	13.6 (3.0)	.14						
5min after adjusting position	13.5 (3.2)	12.8 (3.3)	10.8 (3.3)	<.001	-0.7(-1.5-0)	.04	-2.7(-3.4- -2.0)	<.001	-2.0(-2.7- -1.2)	<.001
Before extubation	11.1 (2.7)	10.3 (2.8)	8.9 (2.3)	<.001	-0.8(-1.4- -0.2)	.003	-2.2(-2.8- -1.7)	<.001	-1.4(-2.0- -0.9)	<.001
**$PaCO_2$ (mmHg)**										
When stitching	47.9 (5.7)	47.4 (5.4)	47.3 (5.3)	.45						
5min after adjusting position	44.7 (5.8)	45.6 (5.7)	45.0 (5.9)	.25						
5min post-extubation	49.8 (6.9)	48.4 (6.6)	45.2 (6.1)	<.001	-1.4(-3.0- 0)	.06	-4.6(-6.1- -3.2)	<.001	-3.2(-4.7- -1.7)	<.001
**PaO_2/FiO_2 (mmHg)**										
When stitching	439.6 (37.1)	446.7 (40.3)	443.3 (38.1)	.13						
5min after adjusting position	444.7 (35.8)	452.2 (38.5)	470.1 (39.5)	<.001	7.5(-1.0- 16.0)	.10	25.4(17.0- 33.8)	<.001	17.9(9.4- 26.3)	<.001
5min post-extubation	324.9 (26.6)	329.8 (30.5)	352.6 (36.7)	<.001	4.9(-2.1- 11.9)	.28	27.7(20.7-34.7)	<.001	22.8(15.8-29.8)	<.001

Abbreviations: Cdyn, dynamic lung compliance (Vt/(Pmax-PEEP); △P, driving pressure (P_{plat}-PEEP); PaO_2, partial pressure of Oxygen; FiO_2, fraction of inspiration Oxygen; MD, mean Difference.

Data are expressed as mean (SD).

© 2024 Wang X et al. *JAMA Network Open*.

eFigure 1. Vital Signs of the Patients

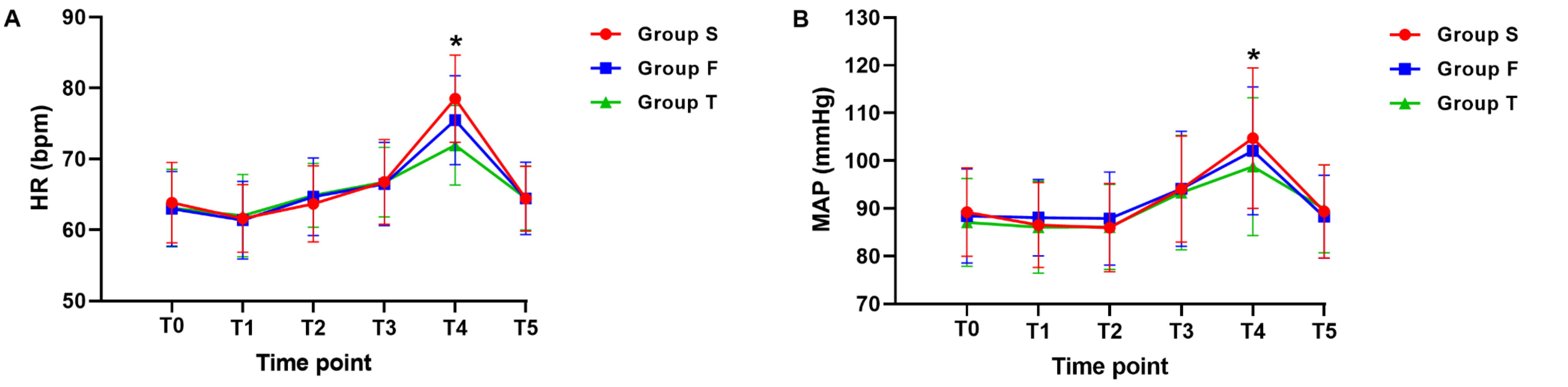

A: Heart rate (beats per minute)
B: Mean Arterial Pressure (mmHg)
T0: the beginning of surgery; T1: the end of surgery; T2: immediately after adjusting position; T3: the moment before extubation; T4: 1 min after extubation; T5: when leaving the Post Anaesthesia Care Unit.
*$P < .017$ Group T compared with Group S and Group F.

© 2024 Wang X et al. *JAMA Network Open*.

eFigure 2. Postoperative Pain of the Patients

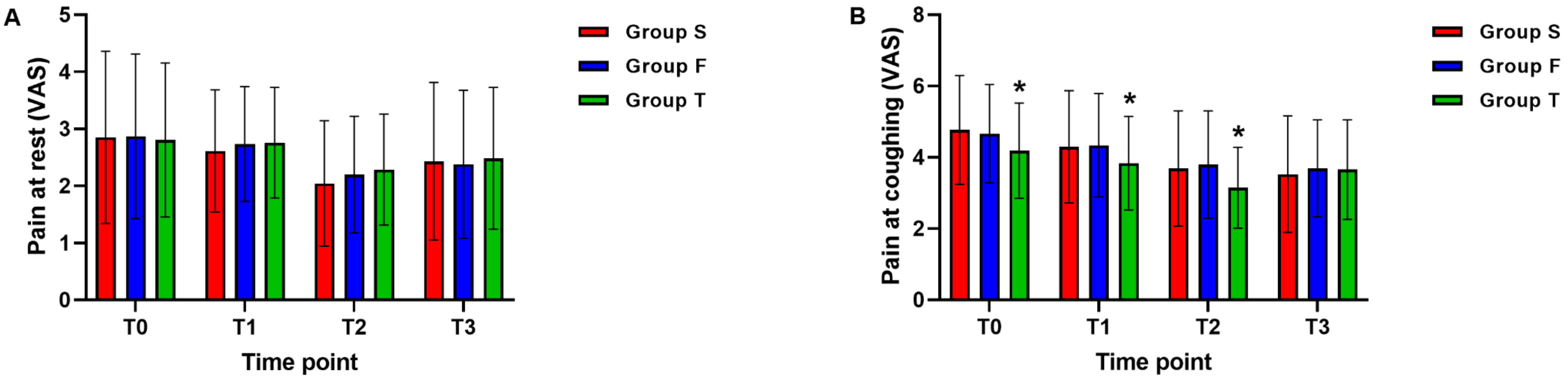

A: Pain at rest
B: Pain while coughing
T0: 5 min after extubation; T1: 30 min after extubation; T2: before leaving Post Anaesthesia Care Unit; T3: postoperative 24 hours.
**P* < .017 Group T compared with Group F and Group S.

© 2024 Wang X et al. *JAMA Network Open*.
