# Supplementary material for: Semirecumbent Positioning During Anesthesia Recovery and Postoperative Hypoxemia: A Randomized Clinical Trial
Source: JAMA Netw Open. 2024 Jun 28;7(6):e2416797. doi: 10.1001/jamanetworkopen.2024.16797 (PMC11214118; doi:10.1001/jamanetworkopen.2024.16797)
Supplement: Supplement 3. — Data Sharing Statement [file jamanetwopen-e2416797-s003.pdf]

Data Sharing Statement

Wang. Semirecumbent Positioning During Anesthesia Recovery and Postoperative Hypoxemia. *JAMA Netw Open*. Published June 28, 2024.
doi:10.1001/jamanetworkopen.2024.16797

Data
Data available: Yes
Data types: Deidentified participant data
How to access data: wangxh_77@163.com
When available: With publication

Supporting Documents
Document types: Statistical/analytic code, Informed consent form
How to access documents: wangxh_77@163.com
When available: With publication

Additional Information
Who can access the data: researchers whose proposed use of the data has been approved
Types of analyses: for any purpose
Mechanisms of data availability: with a signed data access agreement
